# Positively Charged Polymers Based on Cyclodextrins for Trametinib and Selumetinib Delivery in Glioblastoma Cancer

**DOI:** 10.1002/cmdc.202501004

**Published:** 2026-02-15

**Authors:** Noemi Bognanni, Maria Teresa Gentile, Antonia Feola, Valentina Giglio, Martina Dragone, Carla Isernia, Graziella Vecchio

**Affiliations:** ^1^ Dipartimento di Scienze Chimiche Università degli Studi di Catania Catania Italy; ^2^ Department of Environmental Biological and Pharmaceutical Sciences and Technologies University of Campania “Luigi Vanvitelli” Caserta Italy; ^3^ Dipartimento di Biologia Università degli Studi di Napoli “Federico II” Napoli Italy; ^4^ Institute for Polymers, Composites and Biomaterials CNR‐IPCB Catania Italy

**Keywords:** β‐cyclodextrin, brain cancer, drug carriers, polyglutammic acid

## Abstract

Glioblastoma (GB) is the most common and aggressive malignant brain tumor, with a median survival of only 12–15 months despite current treatments with surgery, radiotherapy, and temozolomide (TMZ). Although TMZ induces cytotoxic DNA methylation in tumor cells, its efficacy is often limited by resistance mechanisms. To overcome these limitations, alternative therapeutic strategies—such as targeting the mitogen‐activated protein kinase/extracellular signal‐regulated kinase (MAPK/ERK) signaling pathway with MEK inhibitors like trametinib and selumetinib—are being explored. However, their clinical success is currently hindered by inadequate delivery across the blood–brain barrier and dose‐limiting toxicity. Nanoparticles, particularly positively charged systems, offer enhanced cellular uptake and therapeutic performance due to their strong interactions with negatively charged cell membranes. Cyclodextrin (CyD)‐based polymers are promising systems owing to their low toxicity and ability to form inclusion complexes with drugs. In this work, we investigate two cationic CyD polymers as potential nanocarriers for GB therapy based on trametinib and selumetinib. Their multivalent architecture and positive charge can facilitate both the encapsulation of drugs and membrane interactions. These systems present promising candidates for enhancing the efficacy of GB treatment.

## Graphical abstract

β‐cyclodextrin‐based multicharged polymers—linear or cross‐linked—enhance the delivery of mitogen‐activated protein kinase inhibitors (selumetinib, trametinib) in glioblastoma cells. These carriers enhance the antiproliferative effect of the drugs and strengthen extracellular signal‐regulated kinase pathway inhibition.

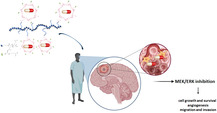

## 1 Introduction

Glioblastoma (GB) is the most common form of brain cancer, accounting for more than 80% of all malignant brain tumors, with the highest rates occurring in older adults, particularly those between 65 and 75 years old [1, 2]. It is associated with a poor prognosis and a median survival time of ≈12–15 months, despite the use of multimodal treatment approaches. Current GB therapy combines maximal safe surgical resection followed by radiotherapy and chemotherapy with temozolomide (TMZ) (the Stupp protocol) [1]. TMZ is an oral alkylating agent that works by adding methyl groups to DNA at specific sites, mainly the O^6^ and N^7^ positions of guanine. This methylation damages the DNA of rapidly dividing tumor cells, leading to faulty replication and cell death, particularly when the tumor lacks effective DNA repair via the O^6^‐methylguanine‐DNA methyltransferase (MGMT) enzymes. However, TMZ also affects healthy dividing cells, especially in the bone marrow, leading to side effects such as myelosuppression (neutropenia, thrombocytopenia, and anemia), nausea, vomiting, fatigue, and an increased risk of infection. Moreover, some tumors produce high levels of the enzyme MGMT, which removes methyl groups and restores the normal DNA structure, effectively undoing the TMZ‐induced DNA damage. Even in tumors initially sensitive to TMZ, resistance can emerge over time as cells lose components of the mismatch repair system, such as MSH6, allowing them to tolerate persistent DNA lesions without triggering apoptosis. Other cells adapt by enhancing alternative DNA repair pathways or by selecting for a more stem‐like, resilient population that can survive chemotherapy. Through these changes, GB gradually becomes less responsive to TMZ, leading to treatment failure and tumor recurrence [2]. To overcome these limitations, alternative therapeutic strategies—such as targeting the mitogen‐activated protein kinase/extracellular signal‐regulated kinase (MAPK/ERK) signaling pathway with MEK inhibitors (MEKi) like trametinib and selumetinib—are being explored. However, their clinical success in glioma has been restricted by poor brain penetration and dose‐limiting toxicity, highlighting the need for advanced delivery systems to fully exploit their therapeutic potential.

In recent years, nanoparticles (NPs) have emerged as effective approaches for delivering a variety of drugs [3, 4]. Particularly positively charged NPs have attracted increasing interest as drug delivery vehicles due to their enhanced interactions with negatively charged cell surfaces, leading to greater uptake, improved endosomal escape, and superior therapeutic outcomes [5].

Recent advances in the design of cationic delivery systems have further refined these benefits. In some cases, pH‐responsive systems have been investigated [6, 7] to trigger drug release under more acidic conditions (pH ∼ 5.0). Additionally, permanently charged groups have been introduced in NPs, such as polyarginine‐based systems, which have been widely studied to facilitate cellular uptake [8, 9, 10].

Among the systems investigated for building NPs, cyclodextrin (CyD) polymers have garnered significant attention due to their low toxicity and ability to form inclusion complexes with a variety of drugs [11, 12, 13, 14]. CyDs, cyclic oligosaccharides composed of glucopyranose units, are widely used in pharmaceutical applications [11, 12, 15] and can be integrated into inorganic or polymeric NPs [13, 16, 17, 18].

Polymeric CyD‐based NPs have been synthesized through various strategies, including the functionalization of linear backbones or the use of crosslinking agents [14, 19, 20], and among these, the commonly used epichlorohydrin (EPI) [21, 22, 23]. The CyD polymers exhibit enhanced properties such as encapsulation capabilities, making them suitable for drug delivery systems. Polymerization can also introduce functional groups into the CyD polymer to enhance its properties, thereby facilitating drug uptake. Notable CyD‐based systems include CALAA‐01, currently in phase II clinical trials as a gene delivery system [24, 25, 26].

**FIGURE 1:**
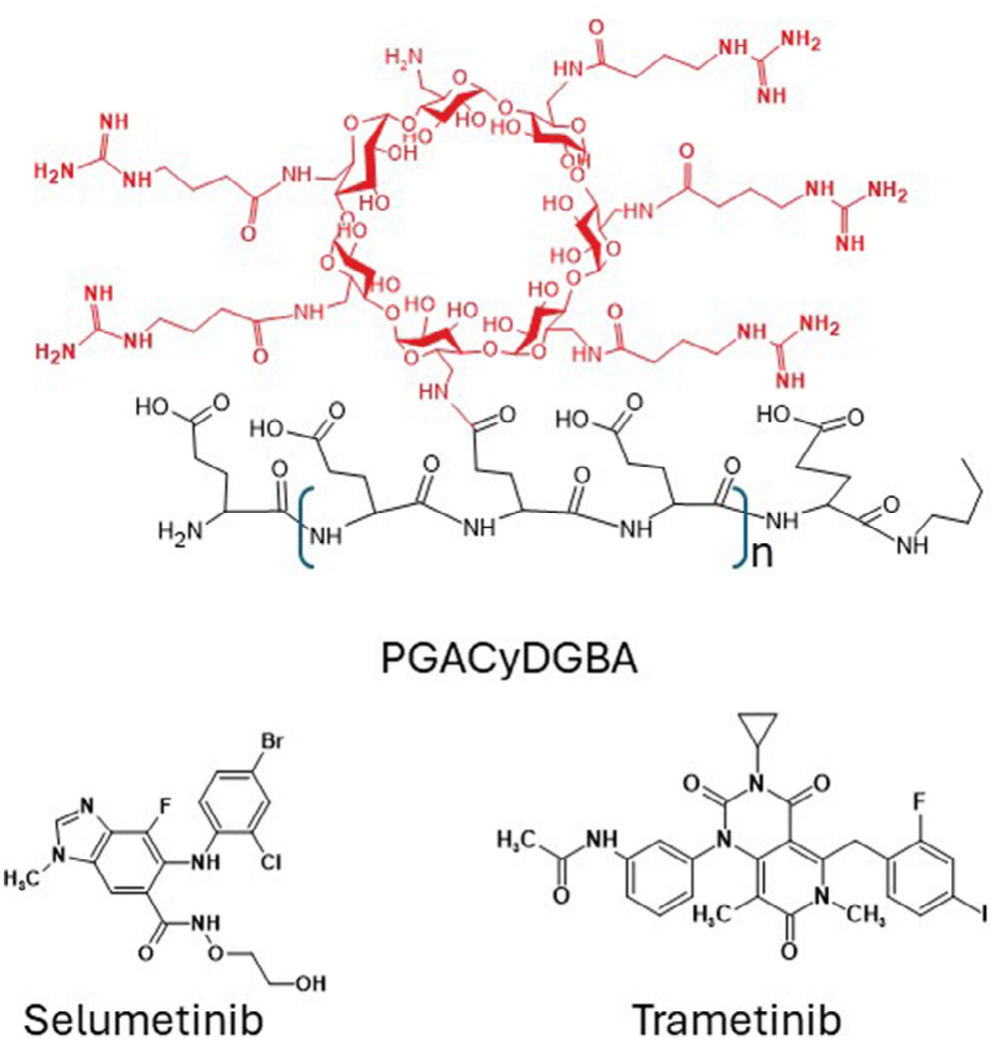
Representative structure of PGACyDGBA and the anticancer drugs investigated.

Building on the advantages of multicavity systems and the importance of NP charge, in this work, we investigated two positively charged polymers based on βCyD. We synthesized a linear, multicharged polymer (PGACyDGBA) with guanidinium groups (Figure 1) by functionalizing the polymeric backbone of polyglutamic acid (PGA) [27] with βCyD prefunctionalized with guanidinobutyric acid (GBA). We also studied a commercially available cross‐linked polymer (QABCyDPS) with positive permanent charges due to quaternary amino groups. Instead, the presence of permanent positive charges, due to guanidinium or quaternary amino groups, in the polymers can facilitate crossing of the blood–brain barrier [28, 29, 30]. Both PGACyDGBA and QABCyDPS were investigated for their ability to deliver trametinib and selumetinib in GB cell lines. These drugs are promising MEKi currently under investigation for the treatment of GB [31, 32]. Indeed, MEKi are being investigated as a new treatment for GB, showing promise in preclinical and clinical studies by targeting the MEK/ERK pathway, which is crucial for cell survival, proliferation, and DNA repair. However, they face challenges due to poor solubility under physiological conditions. Trametinib and selumetinib have aromatic rings in their structure and their interaction with CyDs and enhancing solubility can be hypothesized as reported for other lipophilic drugs with similar structures [33, 34].

The novel CyD polymers investigated in this study aim to address these limitations and improve therapeutic outcomes in GB treatment. In this regard, the trametinib and selumetinib effects were evaluated either alone or in combination with βCyD conjugates on the two GB cell lines U87MG and U251. Despite their common origin in GB, these cell lines exhibit distinct biological features. Indeed, it is well‐known that U87MG cells proliferate and migrate faster than the others under the same culturing conditions, while U251 cells are characterized by an upregulated glycolytic pathway, which supports key cancer cell traits such as adaptation to nutrient‐deprived conditions and resistance to oxidative stress and apoptosis [35]. In addition, U251 cells exhibit constitutive activation of the MEK/ERK pathway, which contributes to their aggressive phenotype and resistance to therapy [36].

We found that CyD polymers improved the solubility of the poorly water‐soluble drugs [31] selumetinib and trametinib and their toxicity against GB cells.

## 2 Results and Discussion

### 2.1 Synthesis and Characterization

The linear multicharged polymer PGACyDGBA was synthesized via condensation reactions from PGA and heptakis 6‐CyDNH_2_, which had been modified with GBA in a previous reaction step. The condensation reactions were carried out under green conditions, using water as the solvent and 4‐(4,6‐dimethoxy‐1,3,5‐triazin‐2‐yl)‐4‐methylmorpholinium chloride (DMTMM) as the activating agent (Figure S1). The condensation reaction gave high yields and enabled the modulation of the number of CyDs in the final product. The final product is a polydisperse system with an average structure similar to that reported in Figure 1.

^1^H NMR spectrum (Figure S2) shows the signals of CyD protons resonate at about 5.0 ppm (H‐1), and between 4.0 and 3.0 ppm (H‐3, ‐6, ‐5, ‐4, and ‐2). Protons of γ*‐*GBA and PGA chains resonate at 3.0 ppm and at 2.0–1.6 ppm. The integral ratios of the CyD Hs‐1 signal and the signal of the CH_3_ butyl protons of PGA allowed for the determination of the average number of CyD units, which functionalized the PGA peptide. This corresponded to 7 CyD units. Moreover, the integral ratio of the signal due to the CH_2_ of GBA at 3.12 ppm and the signals of the *n*‐butyl protons suggested the average number of GBA moieties, corresponding to ≈35 units in the polymer, that is, about five moieties per CyD unit.

Mass spectrometry analysis was additionally performed to further elucidate the structural characterization of the newly synthesized PGACyDGBA. The MALDI‐TOF spectrum acquired in linear mode, reported in Figure S3, shows that the molecular weight of PGACyDGBA is distributed in the m/z range from 6,000 to 24,000. Furthermore, the average mass difference between two consecutive peaks falls in the range of ≈1800–1900 Da, suggesting that the repeat unit consists of a CyD unit bearing five GBA moieties, as expected. The number‐average (Mn) and weight‐average molecular weights (Mw) calculated from the spectrum were 11,290 and 12,800 Da, respectively. However, a discrepancy is observed in the number of CyD units attached to the polymer backbone when comparing the results obtained by ^1^H NMR and MALDI‐TOF analyses. This result may arise from the well‐known difficulty in accurately determining the average molecular weight of polymers with high polydispersity indices by MALDI‐MS [37]. Indeed, several studies have shown that high‐mass components are often under‐represented with respect to lower‐mass species, leading to an underestimation of the average molecular weight [38]. This phenomenon can be attributed to multiple factors, including the choice of matrix; in our case, for example, the use of 2,5‐di‐hydroxybenzoic acid (DHB) instead of sinapic acid (SA) results in an additional shift of the distribution toward lower m/z values (Figure S4).

The size of PGACyDGBA and of the commercially available crosslinked polymer QABCyDPS were investigated by dynamic light scattering (DLS). The hydrodynamic diameters were determined in phosphate buffer and in a 0.5 M NaCl solution to compare the dimensions at different ionic strengths (Figures S5 and S6, Table 1).

**TABLE 1:** Size (Z‐Average, d nm) and Z‐Potential (mV) of PGACyDGBA and QABCyDPS in phosphate buffer (pH 7.4).

Polymer	Size (Z‐Average, d nm)	Z‐Potential, mV
PGACyDGBA	23 ± 2	41 ± 5
PGACyDGBA^a^	7 ± 2	
QABCyDPS	49 ± 3	44 ± 3
QABCyDPS^a^	29 ± 3	

a NaCl 0.5 M in phosphate buffer pH 7.4.

Data reported in Table 1 showed that PGACyDGBA formed smaller NPs than QABCyDPS. The size distribution depended on the presence of NaCl. In water, the NP size of PGACyDGBA is about 23 nm, but it is significantly reduced to 7 nm in NaCl solution. The size variation might be due to the high number of positive charges. Their high electrostatic repulsion forms an elongated structure in water; in the presence of Cl^‐^, the positive charges are shielded, resulting in smaller NPs. The cross‐linked QABCyDPSs have a higher molecular weight and form bigger NPs. The effect of ionic force is less evident, likely due to the cross‐linked structure of the polymer. The Z‐potential of the systems at physiological pH was also evaluated (Table 1). While PGACyDGBA and QABCyDPS showed Z values of +41 ± 5 and +44 ± 3 mV, respectively (Figure S7), consistent with the positive multicharges in the polymers.

**FIGURE 2:**
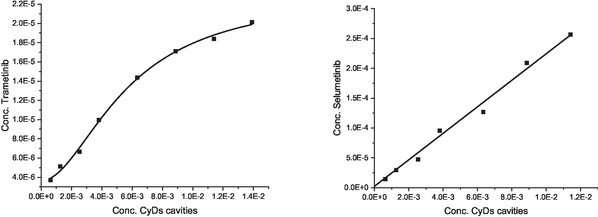
Phase‐solubility diagrams of trametinib (left) and selumetinib (right) with QABCyDPS in phosphate buffer at pH 7.4.

### 2.2 Solubility Experiment

Due to the low solubility of selumetinib and trametinib (≈1.28 × 10^−5^ M and <10^−6^ M, respectively) investigated [31], we examined their solubility with the QABCyDPS polymer, which was the most active in the biological assay. The phase solubility of trametinib and selumetinib in the presence of QABCyDPS polymer was determined at pH 7.4 [39]. The CyD polymers can be considered a multicavity system, and the cavities can be assumed to be equivalent and independent binding sites [40, 41]. The diagram shows the solubility of the drug versus the polymer concentration, expressed as the concentration of CyD units (Figure 2).

**FIGURE 3:**
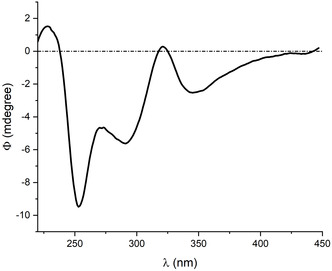
CD spectrum of QABCyDPS/selumetinib in water.

In the case of selumetinib, an A‐type diagram was observed. The linear A‐type profile is indicative of a complex with 1:1 stoichiometry, showing a 20‐fold increase in drug solubility for selumetinib with the crosslinked polymer. In the case of trametinib, an A_*N*_‐type curve was found. The negative deviation from linearity for the A_*N*_‐type can suggest the formation of complexes with different stoichiometry [38, 39]. The solubility of trametinib was five times higher in the presence of QABCyDPS.

The interaction of the drugs with the polymers was also investigated using circular dichroism (CD) spectroscopy (Figure 3) at pH 7.4 in a water/methanol solution. Selumetinib is not a chiral molecule and did not show circular dichroism spectra. Spectra of selumetinib in the presence of the polymer showed induced CD signals in the region of absorption of selumetinib at 346, 291, and 253 nm, suggesting the interaction of the drug with the CyD cavities of the polymer. No induced CD spectra were observed in the case of trametinib, in keeping with the solubility diagram results [42, 43].

### 2.3 Cell Viability

**FIGURE 4:**
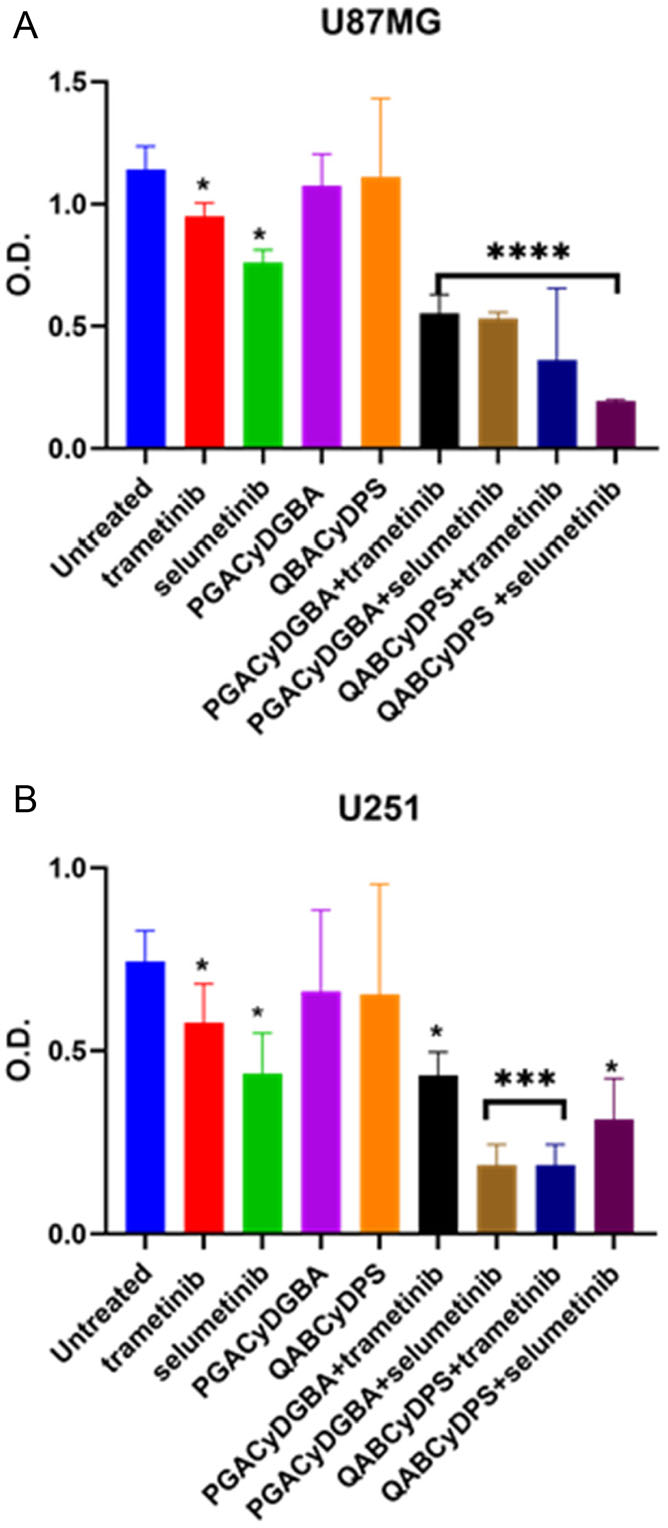
PGACyDGBA and QABCyDPS increase MEKi‐induced impairment of GB cell viability. MTT assay on (A) U87MG and (B) U251 cell lines. Untreated cells were used as a positive control. Error bars are representative of the SD of three independent experiments performed in quadruplicate. The significance of the data obtained was tested by two‐way ANOVA with Sidak's multiple comparisons (****p < 0.0001, ***p < 0.005, *p = 0.02 compared to untreated cells).

The cell viability of two drugs alone and in the presence of PGACyDGBA and QABCyDPS was investigated in U251 and U87MG cell lines. U87MG and U251 are both human GB cell lines widely used in neuro‐oncology research, but they differ in their genetic background and biological behavior. Indeed, this approach better reflects the heterogeneity seen in patient tumors. As shown in Figure 4, treatment with trametinib alone led to a 22% reduction in cell viability in U251 and a 10% reduction in U87MG at 48 h compared to untreated controls. When combined with PGACyDGBA, the reduction in viability increased to 41% in U251 and 50% in U87MG. The combination with QABCyDPS resulted in a 77% decrease in U251 cell viability and a 75% decrease in U87MG cell viability. On the other hand, selumetinib treatment alone resulted in a 41% reduction in U251 and a 25% reduction in U87MG cell viability compared to untreated controls. When combined with PGACyDGBA, cell viability was further reduced to 25% in U251 cells and 50% in U87MG cells, suggesting an enhanced inhibitory effect. In U251 cells, no significant difference in cell viability was observed when selumetinib was combined with QABCyDPS, compared to selumetinib alone, indicating that QABCyDPS does not potentiate the effect of selumetinib under the tested conditions. Meanwhile, in U87MG cells, the combination of selumetinib with QABCyDPS increased cell viability by 33% compared to untreated controls. These results suggest that the combination of MEKi with βCyD polymers enhances their inhibitory effect on the MEK‐dependent signaling pathway.

### 2.4 ERK Phosphorylation Analysis

**FIGURE 5:**
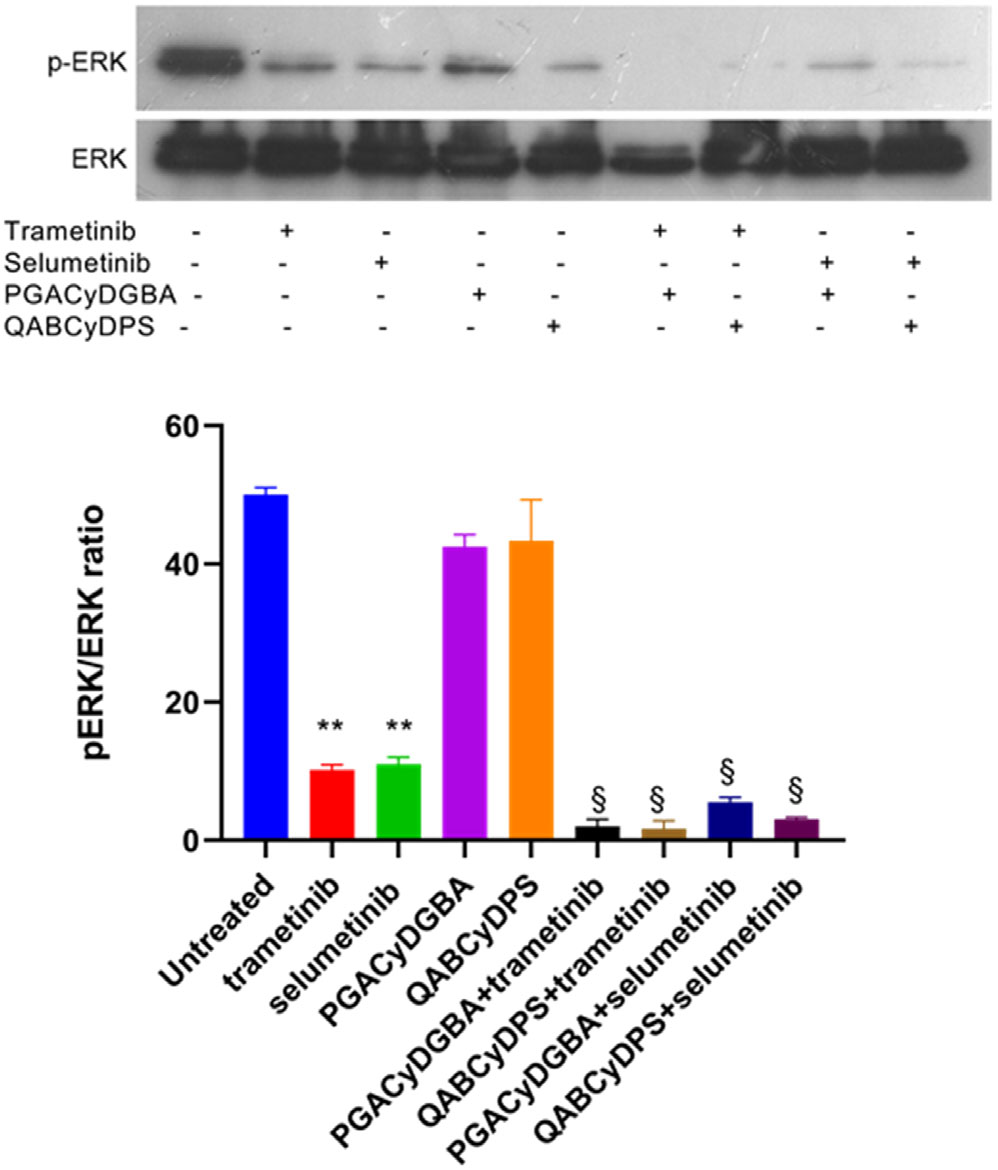
PGACyDGBA and QABCyDPS increase MEKi‐induced inhibition of ERK1/2 phosphorylation in the GB cell line. Western blotting detection of p‐ERK1/2 and ERK1/2 proteins in U251 cell line. The blot is representative of three separate experiments. The graphs show the relative quantification of p‐ERK1/2 and ERK1/2 in the different samples. Data are expressed as ratios of p‐ERK/ERK. **p < 0.01 versus controls. §*p* < 0.001 versus trametinib and selumetinib alone.

MEK phosphorylates and activates its downstream effector ERK, which in turn regulates tumor cell proliferation and survival, angiogenesis, migration, and invasion. For this reason, we analyzed ERK phosphorylation in U251 cells treated with trametinib and selumetinib, both alone and in combination with βCyD conjugates, using Western blotting. As shown in Figure 5, U251 cells exhibit constitutive ERK phosphorylation, even under serum‐free culture conditions, consistent with persistent activation of the MEK pathway. Treatment with trametinib or selumetinib at 1 μM concentration resulted in a significant reduction in ERK phosphorylation, as expected. Notably, the combination of either MEKi with PGACyDGBA and QABCyDPS led to a further significant decrease in ERK phosphorylation, indicating that the polymers potentiate the inhibitory activity of MEKi on the MEK/ERK signaling pathway.

The data presented demonstrate that the combination of MEKi (trametinib and selumetinib) with the two carriers (PGACyDGBA and QABCyDPS) enhances their antiproliferative effects in GB cell lines. Both trametinib and selumetinib significantly reduced cell viability when used alone, consistent with their role as selective inhibitors of the MEK/ERK pathway. However, their efficacy was markedly increased when combined with PGACyDGBA and QABCyDPS polymers, particularly this last in combination with trametinib, which resulted in a dramatic 77% reduction in viability. Western blot analysis further confirmed this cooperative effect at the molecular level. U251 cells displayed constitutive ERK phosphorylation even in the absence of serum, reflecting sustained MEK/ERK pathway activation, a hallmark of GB aggressiveness and therapeutic resistance. Treatment with trametinib or selumetinib alone effectively reduced ERK phosphorylation, but the combination with charged CyD polymers led to further significant suppression of ERK activation. This supports the hypothesis that the polymers enhance MEK inhibition by modulating membrane microdomains (e.g., lipid rafts), potentially improving drug accessibility to the MEK/ERK signaling complex. Moreover, the differential effects observed between PGACyDGBA and QABCyDPS suggest that structural differences among β‐CyDs influence their cooperative action with MEKi. QABCyDPS, which showed the most significant potentiation with trametinib, may possess properties that better facilitate drug delivery, membrane interaction, or intracellular accumulation. Collectively, these findings highlight the therapeutic potential of combining MEKi with the two carriers in GB treatment. Such combinations may overcome intrinsic resistance mechanisms by enhancing MEKi bioavailability or disrupting compensatory signaling networks.

Furthermore, the enhanced cytotoxic effect observed in the presence of the polymers suggests that comparable therapeutic efficacy may be achieved at lower drug doses, potentially reducing side effects.

## 3 Materials and Methods

### 3.1 Materials

The butyl‐polyglutamate (20) sodium salt (3 kDa, PGA), with 20 repeat units and the C‐terminal amino acid amidated with butylamine, was acquired from IRIS Biotech GmbH. Heptakis (6‐deoxy‐6‐amino)‐β‐cyclodextrin heptahydrochloride (6‐CyDNH_2_) was acquired from CycloLab Ltd. Soluble (2‐hydroxy‐3‐N,N,N‐trimethylamino)propyl‐beta‐cyclodextrin polymer crosslinked with EPI (QABCyDPS, 74 kDa, about 45 CyD cavities) was purchased from CarboHyde. Trametinib and selumetinib were acquired by MedChemExpress. 4‐Guanidino butyric acid (GBA) was acquired from Merck. TLC plates (Polygram SIL G/UV254 0.20 mm Marcherey‐Nagel) were used. The detection of the products on the TLC plates was achieved using an anisaldehyde solution (5%) in ethanol. Sephadex G‐15 was used for column chromatography.

### 3.2 NMR Spectroscopy

^1^H and ^13^C NMR spectra were recorded at 25°C with a Varian UNITY PLUS‐500 spectrometer at 499.9 and 125.7 MHz, respectively, using standard pulse programs from the Varian library. 2D experiments were acquired using 1k data points, 256 increments and a relaxation delay of 1.2 s. ^1^H NMR spectra were referred to as the solvent signal.

### 3.3 Mass Spectrometry

MALDI MS analysis was performed using a Bruker Ultraflex Extreme MALDI‐TOF/TOF instrument (Bruker, Bremen, Germany). The FlexControl 3.4 and FlexAnalysis 3.4 software (Bruker, Bremen, Germany) were used to control the instrument and process the MS spectra. The mass spectrometer operated in the linear mode, an accelerating voltage of 20 kV and a delay time of 250 ns were applied, with 1000 laser shots collected for each sample. SA or DHB was used as the matrix, dissolved in water/acetonitrile 1:1 with NaCl 1 mM at a concentration of 20 mg/mL and mixed with the sample dissolved in water (0.3 μM) at a sample‐to‐matrix ratio of 1:5.

### 3.4 Dynamic Light Scattering

DLS measurements were performed at 25°C with a Zetasizer Nano ZS (Malvern Instruments, London, UK) operating at 633 nm (He‐Ne laser). The mean hydrodynamic diameter (d) of the NPs was calculated from the intensity measurement after averaging the five measurements. The samples (2 mg/mL) were diluted in phosphate buffer (pH 7.4, 1 mM) prepared with ultrapure, filtered water (0.2 µm filter).

### 3.5 UV–Visible and Circular Dichroism Spectroscopy

UV–vis spectra were recorded with an Agilent Cary 8500 spectrophotometer equipped with a Peltier cell holder. Circular dichroism measurements were performed with a JASCO J‐1500 spectropolarimeter. The spectra were recorded at 25°C on freshly prepared water solutions, pH = 7.4.

### 3.6 Solubility Studies

Trametinib and selumetinib (5 µL, 0.021 M, DMSO solution) were added to 0.120 mL of eight solutions of the CyD polymers in phosphate buffer (100 mM, pH 7.4) at different concentrations as reported elsewhere [42]. The suspensions formed due to the drug precipitation at 7.4 pH were sonicated for 10 min and incubated at 25°C in the dark. After 18 h, the suspensions were centrifuged at 10,800 rpm for 10 min at 25°C. The selumetinib and trametinib concentrations were acquired in the supernatant with UV/vis spectroscopy at the wavelength of maximum absorbance (*λ*_max_) 260 and 330 nm, respectively. A linear calibration plot for free drugs in phosphate buffer at pH 7.4 was previously obtained to determine the molar absorptivity *ε*, with values of 52,841.56 and 23,036.72095 (mol^−1^ L cm^−1^), respectively.

### 3.7 MTT Assay

Cell viability in response to treatment with MEKi (trametinib and selumetinib), β‐CyD polymers (PGACyDGBA and QABCyDPS) alone, and in combination with the drugs (molar ratio PGACyDGBA/drug 1:7 and QABCyDPS/drug 1:45), was assessed using the thiazolyl blue tetrazolium bromide (MTT) assay, as previously described in Ref. [43] U87MG and U251 cells (2.5 × 10^3^ cells/well) were seeded in 96‐well plates and treated with various concentrations of the compounds for 48 h at 37°C in a 5% CO_2_ incubator. Untreated cells were used as a control. Following treatment, 10 µL of MTT solution (5 mg/mL in PBS; Invitrogen–Life Technologies, Eugene, OR, USA) was added to each well and incubated for 3 h at 37°C. After incubation, 100 µL of stop solution was added to each well. Optical density (OD) was measured at 595 nm using a microplate reader (Synergy HTX, BioTek). Each experiment was independently repeated three times, with treatments performed in quadruplicate. Statistical analysis and graphical representations were generated using GraphPad Prism 8.0 (GraphPad Software). Bar plots present mean OD values ± standard deviation (SD).

### 3.8 Western Blot Analysis of ERK Phosphorylation

ERK phosphorylation was assessed by Western blotting. Cytosolic proteins were isolated by scraping cells into a lysis buffer containing 25 mM Tris‐HCl (pH 7.4), 1 mM EDTA, 1 mM EGTA, 0.1 mM NaF, and 0.1 mM Na_3_VO_4_. Protein concentrations were determined using the Bradford assay. Equal amounts of protein (20 µg per sample) were separated by 10% SDS‐polyacrylamide gel electrophoresis and transferred to nitrocellulose membranes. Membranes were blocked at room temperature for 1 h in 5% nonfat milk in tris‐buffered saline (TBS)‐T (TBS with 0.1% Tween 20) and then incubated overnight at 4°C with a primary antibody against phosphorylated ERK (P‐ERK; E‐4, sc‐7383, Santa Cruz Biotechnology) diluted 1:1000 in 3% BSA in TBS‐T. After washing, membranes were incubated with peroxidase‐conjugated anti‐rabbit IgG (1:5000; Amersham Biosciences) for 1 h at room temperature. Detection was performed using an enhanced chemiluminescence (ECL) kit (Millipore). To normalize protein loading, membranes were stripped and reprobed with an antibody against total ERK (9102, Cell Signaling Technology) diluted in TBS‐T. After 1 h of incubation at 25°C, membranes were treated with peroxidase‐conjugated anti‐mouse IgG (1:10,000 in TBS‐T) and visualized as described above. Band intensities were quantified by scanning densitometry using Scion Image software, version 4.5 (Scion Corp., Frederick, MD, USA).

### 3.9 Statistical Analysis

All data are represented as histograms showing the mean ± SD from three independent experiments. Data expressed as the ratio between phosphorylated and total amounts are normalized compared to the control and were tested for significance using the Student t‐test for paired data. All statistical analyses were performed using GraphPad Prism 8.0 (GraphPad Software, Inc., La Jolla, CA, USA), with a *p*‐value ≤0.05 as the cut‐off for statistical significance between groups.

### 3.10 Synthesis of PGACyDGBA

6‐CyDNH_2_ (143 mg, 0.100 mmol) was solubilized in water (1 mL), and the pH of the solution was adjusted to 8.0. DMTMM (143 mg, 0.500 mmol) and GBA (30 mg, 0.20 mmol) were added to the reaction mixture three times under stirring over 2 h. After 24 h, PGA (20 mg, 7,0 μmol) was added under stirring to the reaction mixture. Three aliquots of DMTMM (38 mg, 0.10 mmol) each were added to the solution within 1 h. The reaction mixture was stirred at 25°C for 24 h.

The reaction mixture was purified by Sephadex G‐15 column chromatography, eluted with water. The eluted fractions were spotted on TLC, and the fractions that were positive to the anisaldehyde reagent were dried, and NMR spectra were recorded. 25 mg of the main product, corresponding to the PGA‐CyD conjugate, was obtained.

^1^H NMR (500 MHz, D_2_O) δ(ppm): 4.97 (m, H‐1 of CyD), 4.80–3.20 (m, H‐3, ‐6, ‐5, ‐4, of CyD, CH_2_ PGA), 3.12 (m, α‐CH_2_ GBA), 2.50–1.70 (m, β‐CH_2_ and α‐CH_2_ PGA, β and γ‐CH_2_ GBA), 1.36 (m, CH_2_ butyl chain of PGA GBA), 1.20 (m, CH_2_ butyl chain of PGA), and 0.78 (m, CH_3_ butyl chain of PGA).

Dimension (DLS, Z‐Average) d: 23 ± 2 nm, Zeta potential: 41 ± 5 mV (pH = 7.4).

## 4 Conclusion

We synthesized a multicharged linear polymer based on βCyDs, using PGA as the backbone, and compared this system with a multicharged cross‐linked polymer. Here, we investigated the behavior of these two positively charged polymeric NPs as drug carriers for selumetinib and trametinib, MEK inhibitors that are currently used in clinical trials for the treatment of GB. However, their clinical success has been restricted by poor brain penetration and dose‐limiting toxicity.

We found that combining trametinib and selumetinib with βCyD‐based carriers significantly enhances their antitumor effects in GB cell lines. This synergy results in stronger suppression of the ERK pathway and reduced cell viability, particularly when trametinib is combined with the cross‐linked polymer. Altogether, these data suggest that the carriers likely enhance drug access by modifying membrane microdomains, increasing intracellular drug accumulation, modulating signaling activity, and potentially altering cell sensitivity to treatment as a result of altered membrane organization. From a translational perspective, optimizing carrier design may lead to more efficient and targeted delivery systems, which could contribute to the development of more effective combination therapies and personalized treatment strategies for GB.

## Supporting Information

Additional supporting information can be found online in the Supporting Information Section. **Supporting Fig. S1:** Synthetic scheme for PGACyDGBA polymer. **Supporting Fig. S2:** ^1^H NMR spectrum of PGACyDGBA (D2O, 500 MHz). **Supporting Fig. S3:** MALDI‐TOF MS spectrum of PGACyDGBA acquired in linear mode with SA as matrix. **Supporting Fig. S4:** Overlay of the MALDI‐TOF spectra acquired using SA (blue trace) and DHB (red trace) matrices, highlighting the loss of high‐mass information in the DHB spectrum (green boxed region). **Supporting Fig. S5:** Intensity Size Distribution (DLS) PGACyDGBA in phosphate buffer (red) and PGACyDGBA in NaCl (0.050 M) (green). **Supporting Fig. S6:** Intensity Size Distribution (DLS) QABCyDPS in phosphate buffer (red) and QABCyDPS in buffer and NaCl 0.010 M (green). **Supporting Fig. S7:** Zeta Potential (mV) PGACyDGBA and QABCyDPS.

## Funding

This study was supported by Ministero dell'Università e della Ricerca (SPlat‐G, Grant 2022JXSA9C).

## Conflicts of Interest

The authors declare no conflicts of interest.

## Supporting information

Supplementary Material

## Data Availability

The data that support the findings of this study are available from the corresponding author upon reasonable request.
