# Supplementary material for: Positively Charged Polymers Based on Cyclodextrins for Trametinib and Selumetinib Delivery in Glioblastoma Cancer
Source: ChemMedChem. 2026 Feb 15;21(3):e202501004. doi: 10.1002/cmdc.202501004 (PMC12906940; doi:10.1002/cmdc.202501004)
Supplement: Supplementary file 1 — Supplementary Material [file CMDC-21-e202501004-s001.pdf]

Positively charged polymers based on cyclodextrins for *trametinib* and *selumetinib* delivery in glioblastoma cancer

Noemi Bognanni[a]#, Maria Teresa Gentile[b]#, Antonia Feola[c], Valentina Giglio[d], Martina Dragone[b], Carla Isernia[b] and Graziella Vecchio[a]*

[a] PhD. N. Bognanni, Prof. G. Vecchio
Dipartimento di Scienze Chimiche, ,
Università degli Studi di Catania
Viale A. Doria 6, 95125 Catania, Italy
E-mail: gr.vecchio@unict.it

[b] PhD M. T. Gentile, M. Dragone, Prof. C. Isernia
Department of Environmental, Biological and Pharmaceutical Sciences and Technologies
University of Campania “Luigi Vanvitelli
Viale A. Lincoln 5, 81100 Caserta, Italy

[c] A. Feola
Dipartimento di Biologia,
Università degli Studi di Napoli “Federico II”
Via Vicinale Cupa Cintia 80126 Napoli, Italy

[d] PhD V. Giglio
Institute for Polymers, Composites, and Biomaterials CNR-IPCB,
Via Paolo Gaifami 18, Catania 95126, Italy

#These authors contributed equally to this work

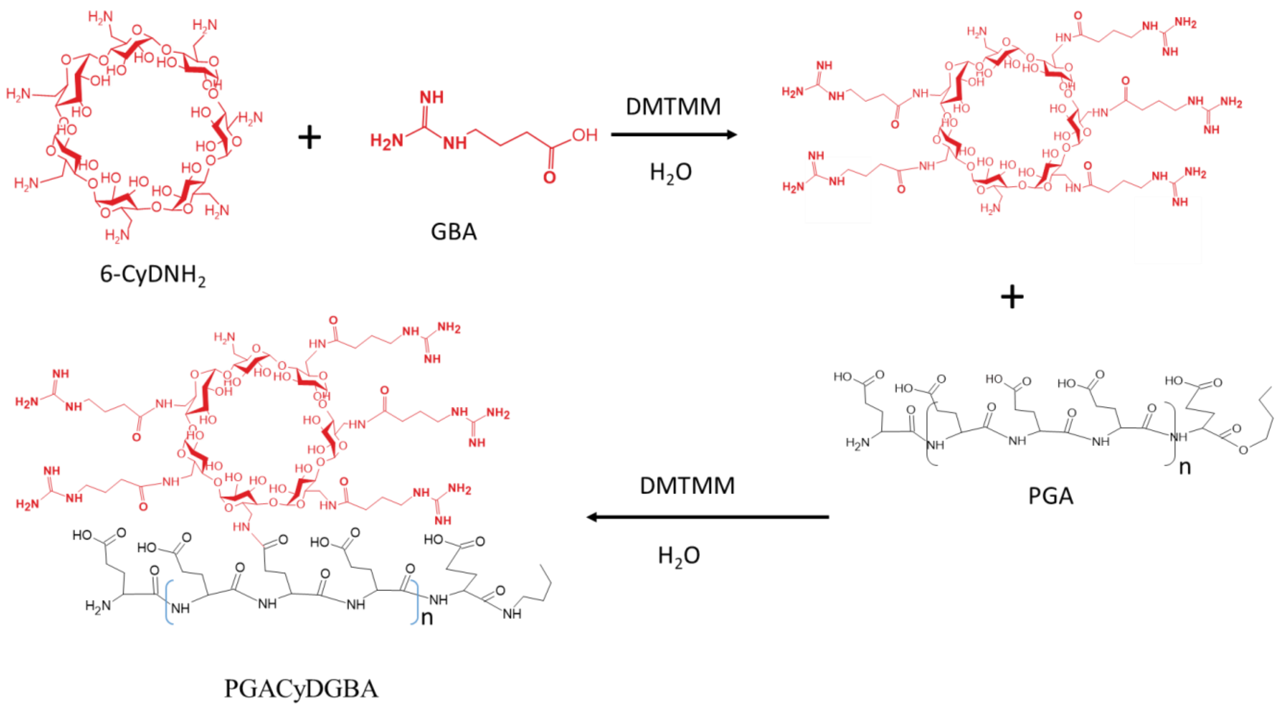

Figure S1. *Synthetic scheme for PGACyDGBA polymer*

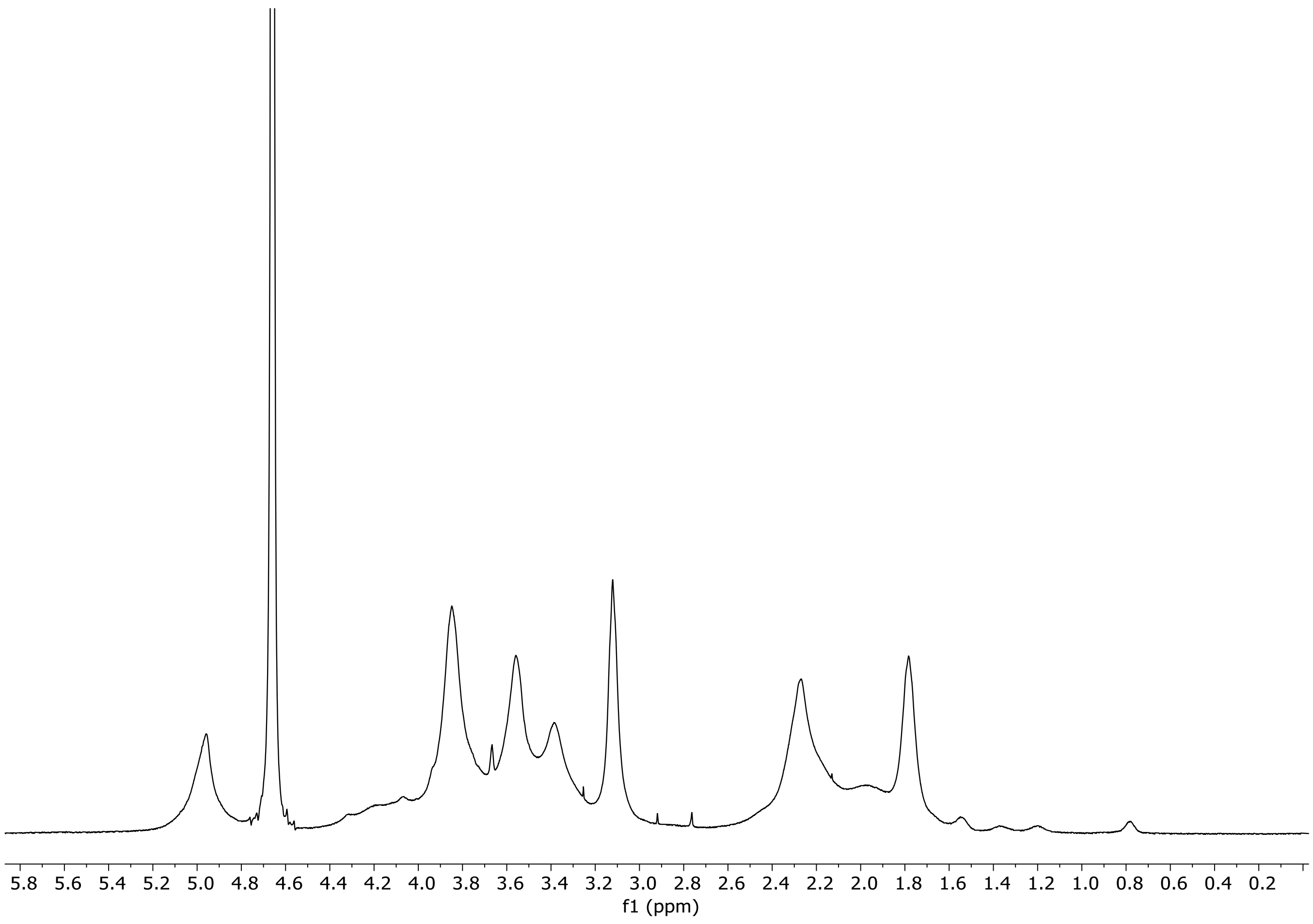

Figure S2. *^{1}H NMR spectrum of PGACyDGBA (D_2O, 500 MHz).*

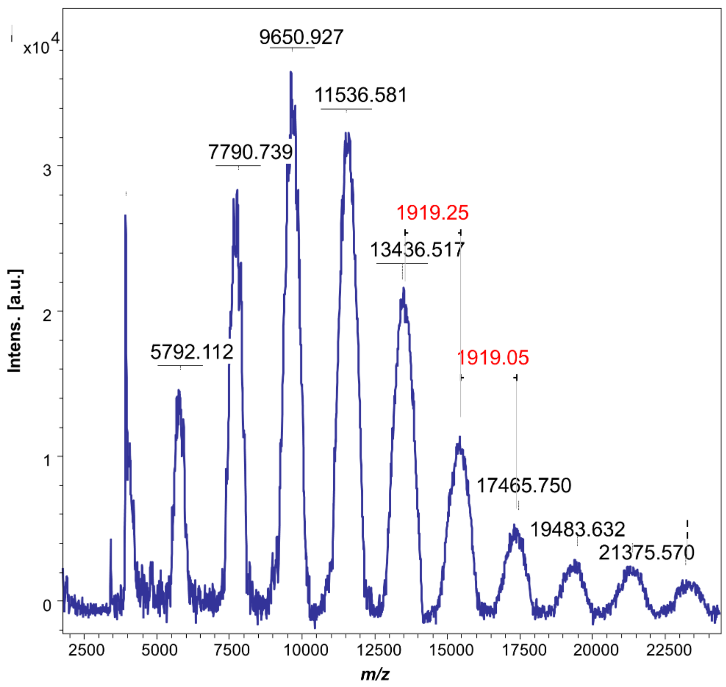

Figure S3. *MALDI-TOF MS spectrum of PGACyDGBA acquired in liner mode with SA as matrix***.**

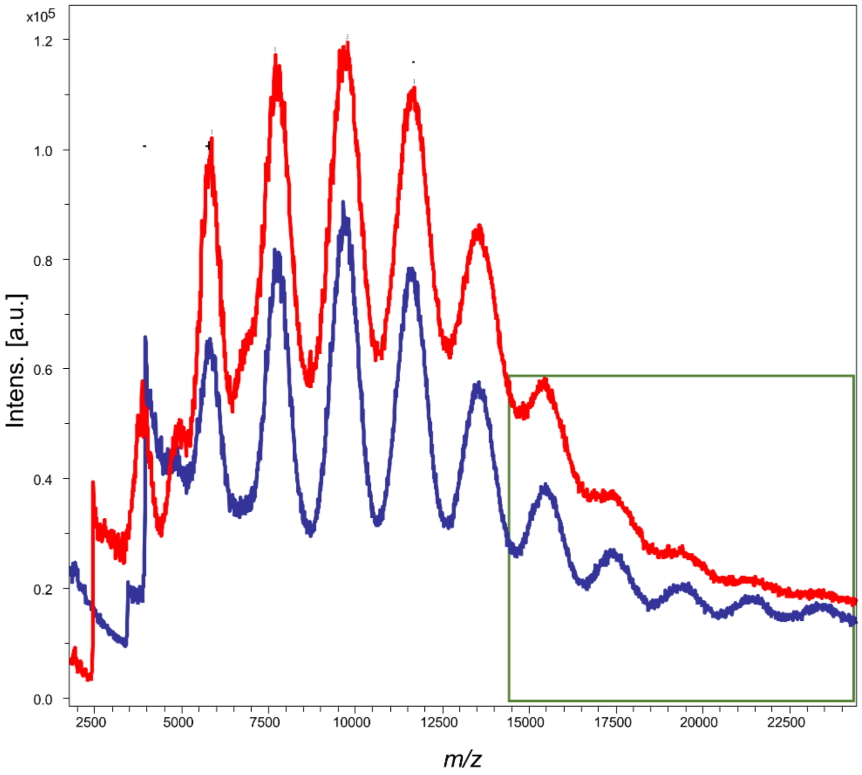

Figure S4. *Overlay of the MALDI-TOF spectra acquired using SA (blue trace) and DHB (red trace) matrices, highlighting the loss of high-mass information in the DHB spectrum (green boxed region).*

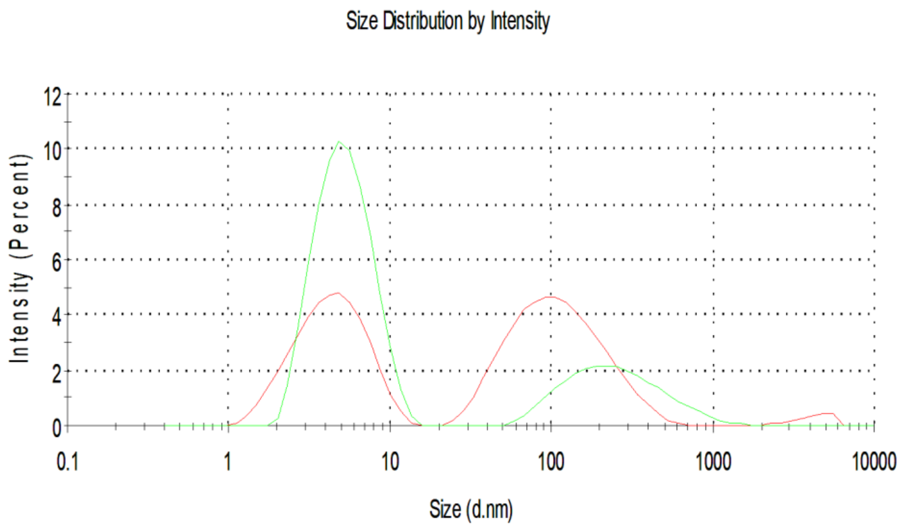

Figure S5. *Intensity Size Distribution (DLS) PGAβCyDGBA in phosphate buffer (red) and PGAβCyDGBA in NaCl (0.050 M) (green)*

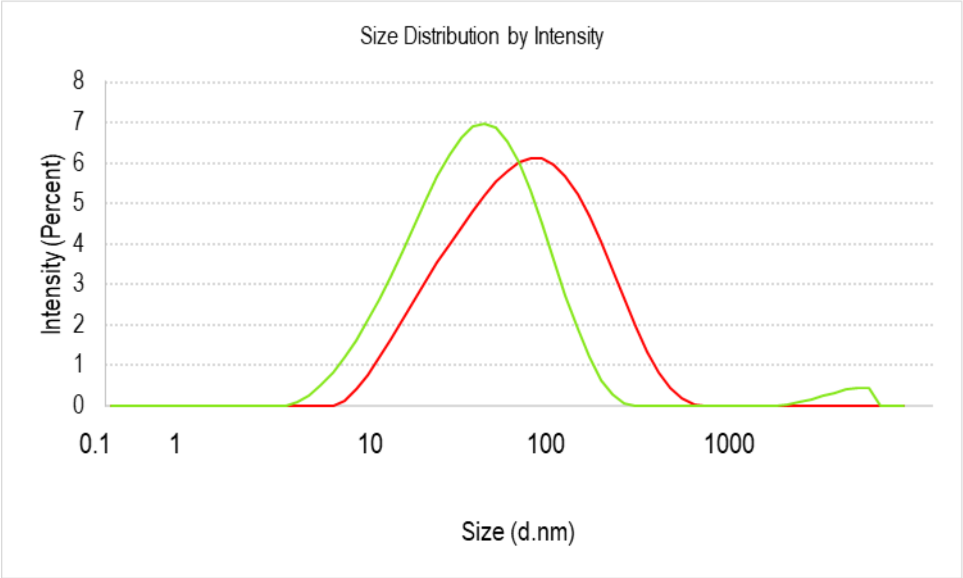

Figure S6. *Intensity Size Distribution (DLS) QABCyDPS in phosphate buffer (red) and QABCyDPS in buffer and NaCl 0.010 M (green)*

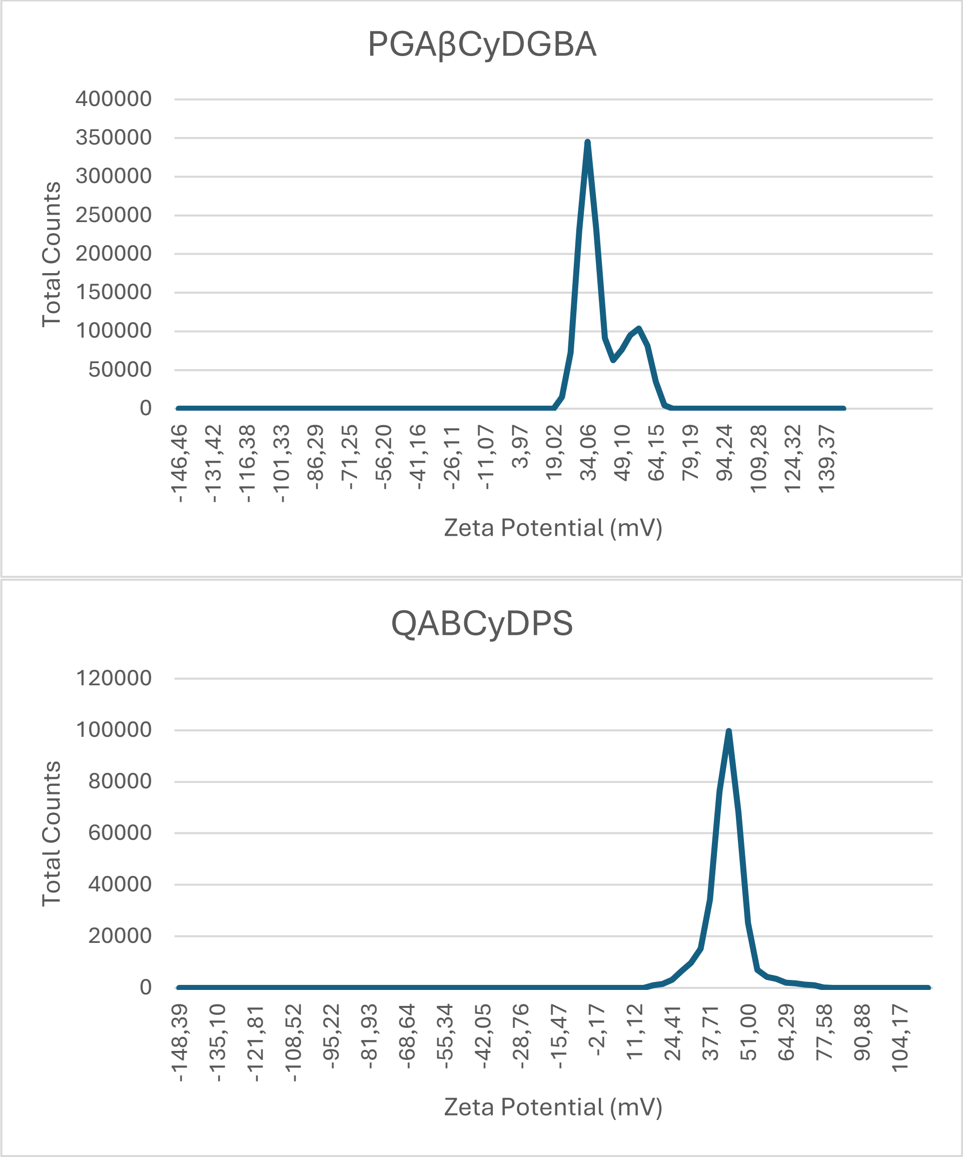

Figure S7. Zeta Potential (mV) PGACyDGBA and QABCyDPS
